# Liver-Targeted AAV-DJ-hCBS Therapy Achieves Long-Term Correction of Metabolic Imbalance in CBS-Deficient Mice

**DOI:** 10.3390/ijms27073338

**Published:** 2026-04-07

**Authors:** Christian P. Joschko, Chih-Chieh Wang, Azuwerus van Buiten, Maaike Goris, Femke Hoogstra-Berends, Joy Wang, Jan Henric Bacurio, Yinxing Chen, Nannan Jia, June Deng, Shiliang Hu, Mariana Nacht, Matthew J. Chiocco, Robert H. Henning, Leo E. Deelman

**Affiliations:** 1 Clinical Pharmacy and Pharmacology, University Medical Center Groningen, Hanzeplein 1, 9713 GZ Groningen, The Netherlands; c.p.joschko@umcg.nl (C.P.J.);; 2 Alexion Pharmaceuticals, AstraZeneca Rare Disease, Genomic Medicine, 100 Binney Street, Cambridge, MA 02142, USA

**Keywords:** homocystinuria, cystathionine β-synthase, transsulfuration, gene therapy, adeno-associated virus, methylation, hyperhomocysteinemia, multi-omics

## Abstract

Cystathionine β-synthase (CBS) deficiency causes classical homocystinuria with severe hyperhomocysteinemia (HHcy) that is inadequately controlled by current therapies. We tested whether liver-targeted CBS gene therapy provides durable biochemical and phenotypic rescue. Using a Cre-inducible adult mouse model of whole-body CBS loss, a single intravenous dose of AAV-DJ-hCBS (3 × 10^12^ or 3 × 10^13^ vg/kg) was administered, and the animals were followed for 12 months. Vector biodistribution showed ~100-fold hepatic enrichment over the kidney and spleen. Both doses rapidly normalized plasma homocysteine (<8 µM), maintaining correction throughout the study while preventing alopecia, weight loss, and loss of adiposity. Liver histology showed resolution of inflammation, and only 2 of 19 mice developed anti-hCBS antibodies. Liver proteomics (3998 proteins quantified) revealed CBS deficiency-associated suppression of tRNA aminoacylation and dysregulation of lipid and carbon metabolism with an HNF4A transcriptional signature, all normalized by therapy. Liver metabolomics demonstrated accumulation of S-adenosylmethionine and S-adenosylhomocysteine and disruption of phosphatidylcholine synthesis, also corrected by treatment. Plasma metabolomics revealed systemic disturbances fully normalized by hepatic CBS restoration. These findings identify the liver as the central metabolic control point in CBS deficiency and support liver-targeted gene therapy as a durable corrective strategy.

## 1. Introduction

Classic homocystinuria (HCU) is an autosomal recessive metabolic disorder caused by loss-of-function mutations in cystathionine β-synthase (CBS), a pyridoxal 5′-phosphate (PLP)-dependent enzyme in the transsulfuration pathway [1]. Affected individuals exhibit multisystem complications, including severe vascular disease, cognitive impairment, skeletal abnormalities, and premature mortality [2,3,4,5].

Current therapies remain limited to dietary methionine restriction, cofactor supplementation (vitamins B6, B12, and folate), and in some cases, betaine to enhance Hcy remethylation. However, these treatments only lower but do not normalize homocysteine levels, which keeps patients at risk for cardiovascular (CV) disease. Particularly pyridoxine-nonresponsive patients have high CV risk due to lower treatment efficacy [1,6,7]. Experimental approaches, such as PEGylated enzyme replacement [8], pharmacological chaperones [9], and early gene therapy strategies, have shown partial homocysteine reduction in preclinical models, but none have achieved durable or comprehensive metabolic correction [10,11].

Normally, homocysteine (Hcy) is cleared either by remethylation or transsulfuration, the latter initiated by CBS. Loss of CBS function disrupts transsulfuration, causing severe hyperhomocysteinemia (HHcy), often exceeding 100 µM. Elevated plasma Hcy contributes to endothelial dysfunction, neurocognitive deficits, and systemic metabolic disturbances, including lowered body fat mass [2,6,12,13,14,15].

Over the past decade, the view of HHcy pathophysiology has expanded from homocysteine toxicity and oxidative stress to recognition of HHcy as a multi-system metabolic disorder with tissue-specific effects. Murine and cellular models have advanced our understanding considerably. For instance, complete CBS knockout in mice produces early postnatal lethality, while models with residual enzyme activity such as the I278T mouse model revealed that CBS deficiency leads to sulfur metabolite imbalance, impaired protein persulfidation, and sphingolipid dysregulation. While in this model, methionine restriction corrects sulfur metabolite disturbances and restores protein persulfidation, thereby extending survival, it does not normalize growth, longevity, or vascular and connective tissue abnormalities [7,16,17,18,19]. Proteostasis-focused studies further show that CBS variants are unstable, with pyridoxine responsiveness linked to partial rescue of folding and stability [18,20,21]. Despite these advances, CBS deficiency alters multiple metabolic pathways beyond sulfur metabolism, including one-carbon metabolism, redox balance, and lipid homeostasis, but the links between these biochemical changes and the clinical manifestations remain poorly defined.

Because CBS is most highly expressed in the liver, this organ represents a logical target for therapeutic intervention. We hypothesized that hepatocyte-specific restoration of CBS function via adeno-associated virus DJ-type (AAV-DJ)-mediated gene therapy normalizes Hcy levels and ameliorates disease phenotypes [22]. Using a Cre-inducible *Cbs*-knockout adult mouse (*Cbs*^-/-^) model, we evaluated the therapeutic potential of liver-targeted delivery of human CBS (AAV-DJ-hCBS) [23,24,25]. Here, we demonstrate that a single i.v. administration of liver-targeted AAV-DJ-hCBS reverses HHcy in the long term, normalizes metabolic balance, and improves disease-associated phenotypes, highlighting liver-targeted gene therapy as a viable path toward a curative treatment for HHcy.

## 2. Results

### 2.1. AAV-DJ-hCBS Normalizes Phenotype and Homocysteine Levels

In the present study, we evaluated the effects of two dose levels of a single injection of liver-targeted AAV-DJ-hCBS therapy at 3 × 10^12^ and 3 × 10^13^ vg/kg body weight, selected to achieve hepatic CBS expression within physiological range or at supraphysiological levels, respectively (*Cbs*^-/-^ + hCBS^low^ and *Cbs*^-/-^ + hCBS^high^). We induced CBS knockout at adulthood (10 weeks of age) using our Cre-inducible *Cbs*^-/-^ mouse, which subsequently exhibits severe HHcy typically exceeding 200 µM (<4 µM in WT), growth retardation, and facial alopecia [26]. In a long-term follow-up study, we treated *Cbs*^-/-^ mice with AAV-DJ-hCBS at 12 weeks and evaluated a 48-week period by assessing effects on phenotype and metabolic profile.

**Figure 1:**
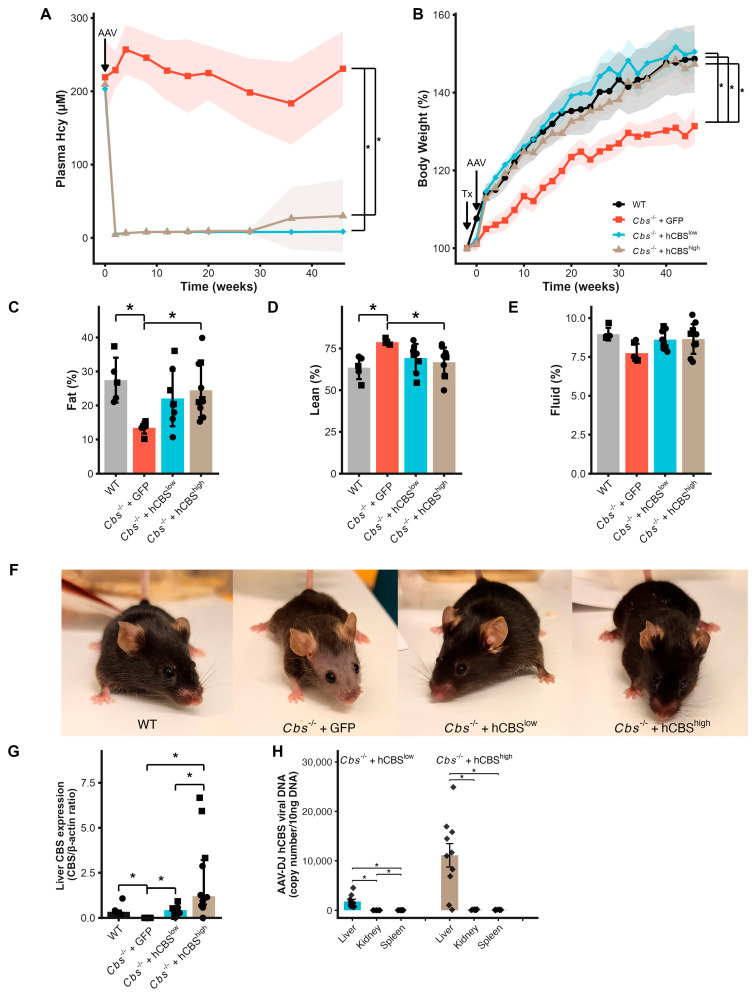
Liver-targeted AAV-DJ-hCBS corrects hyperhomocysteinemia and systemic phenotypes in *Cbs*^-/-^ mice. (**A**) Longitudinal plasma homocysteine (Hcy) concentrations in mice following tamoxifen-induced Cbs deletion (Tx) and AAV-DJ administration. Lines show group means with 95% confidence interval. (**B**) Time course of body weight (percentage of baseline) represented as group mean ± SEM. (**C**–**E**) Body composition at 48 weeks: fat mass (**C**), lean mass (**D**), and fluid mass (**E**) as percentage of body weight. (**F**) Representative images, at 48 weeks, illustrating facial alopecia in *Cbs*^-/-^ + GFP and its prevention by AAV-DJ-hCBS treatment. (**G**) Hepatic CBS expression normalized for beta-actin expression was similar to WT in *Cbs*^-/-^ + hCBS^low^, whereas *Cbs*^-/-^ + hCBS^high^ showed approximately 5-fold higher CBS expression than WT; (**H**) hCBS vector genome copy number per 10 ng genomic DNA in liver, kidney, and spleen. Values are normalized to the median liver value of the *Cbs*^-/-^ + hCBS^high^ group (set to 1). If not otherwise stated, data are represented as mean ± SEM. Circle = male mouse. Square = female mouse. Diamond = sex unknown. Data are from WT (n = 7; black), *Cbs*^-/-^ + GFP (n = 8; red), *Cbs*^-/-^ + hCBS^low^ (n = 9; blue), and *Cbs*^-/-^ + hCBS^high^ (n = 10; beige) mice. * *p*-value < 0.05.

Liver-targeted AAV-DJ-hCBS therapy normalizes *Cbs*^-/-^ phenotypes. To confirm the efficiency and liver specificity of AAV-DJ-mediated transgene delivery, we first assessed vector distribution and hepatic CBS expression. Vector copy numbers measured by ddPCR were ~100-fold higher in the liver compared with the kidney and spleen for both doses, confirming hepatocyte-selective transduction (Figure 1H). Western blotting showed that low-dose AAV-DJ (*Cbs*^-/-^ + hCBS^low^) restored hepatic CBS expression, whereas the high dose (*Cbs*^-/-^ + hCBS^high^) resulted in a further increase (~5-fold) (Figure 1G).

Having established efficient and liver-selective CBS expression, we next examined whether this was sufficient to correct the metabolic consequences of CBS deficiency. Liver-targeted AAV-DJ-hCBS resolved HHcy in CBS-knockout mice within two weeks after injection and remained effective throughout the experiment (Figure 1A). Plasma concentrations of Hcy dropped below the detection limit (<6–8 µM) in both *Cbs*^-/-^ + hCBS^low^ and *Cbs*^-/-^ + hCBS^high^, whereas HHcy persisted in *Cbs*^-/-^ + GFP (AAV-DJ-GFP control) mice. Hcy levels remained undetectable in the treatment groups over the course of the study, except for one mouse in the *Cbs*^-/-^ + hCBS^high^ (mouse 13), which reverted to HHcy at week 38 post-AAV-DJ-hCBS injection.

AAV-DJ-hCBS therapy fully corrected the growth and body composition defects characteristic of CBS deficiency. Mice in the *Cbs*^-/-^ + GFP group showed impaired growth compared to wild-type Cbs^+/+^ mice (Figure 1B). In contrast, the *Cbs*^-/-^ + hCBS^low^ and the *Cbs*^-/-^ + hCBS^high^ groups showed a considerable gain in body weight immediately after AAV-DJ-hCBS injection, effectively restoring their growth curve to that of wild-type mice. Lower body weight in *Cbs*^-/-^ + GFP was due to a lower body fat percentage (13.5 ± 0.7%; Figure 1C) compared to wild-type mice (27.5 ± 2.9%), which was normalized in both *Cbs*^-/-^ + hCBS^low^ and the *Cbs*^-/-^ + hCBS^high^ (22.1 ± 2.9 and 24.5 ± 2.5%, respectively). Consequently, AAV-DJ-hCBS therapy also normalized fluid mass and lean body weight (Figure 1D,E).

All *Cbs*^-/-^ + GFP started developing facial alopecia at approximately 2–3 months after induction of HHcy, resulting in severe facial alopecia at the end of the study (Figure 1F). AAV-DJ-hCBS therapy completely prevented the development of alopecia. Moreover, prevention of alopecia was not due to CBS transgene expression in skin, as CBS expression in skin was low to undetectable in all three groups (*Cbs*^-/-^ + GFP, *Cbs*^-/-^ + hCBS^low^ and *Cbs*^-/-^ + hCBS^high^; Supplemental Figure S3).

In line with our previous study [26], we did not observe major abnormalities in organ weights and blood parameters after termination of the mice (Table 1). However, blood glucose was significantly decreased in *Cbs*^-/-^ + GFP (9.7 ± 1.4 mM) compared to wild-type mice (13.4 ± 1.2 mM). These values were effectively corrected by AAV-DJ-hCBS treatment, as blood glucose was normalized in *Cbs*^-/-^ + hCBS^low^ and *Cbs*^-/-^ + hCBS^high^ (12.8 ± 3.3 and 13.2 ± 0.9 mM, respectively). In addition, the number of red blood cells (RBCs) was decreased in *Cbs*^-/-^ + GFP (8.4 ± 3.3 × 10^12^/L) compared to wild-type mice (12.4 ± 1.4 × 10^12^/L). Again, AAV-DJ-hCBS treatment corrected these values, as RBC was normalized in *Cbs*^-/-^ + hCBS^low^ and *Cbs*^-/-^ + hCBS^high^ (11.4 ± 1.4 and 12.1 ± 1.7 × 10^12^/L, respectively). A similar pattern was observed for the related parameters of hematocrit and total hemoglobin and for the amount of hemoglobin in red blood cells (MCH) (Table 1). Male and female mice responded similarly to AAV-DJ-hCBS treatment, with no sex-dependent differences in HHcy, body weight, or body composition parameters. Collectively, these findings demonstrate that liver-targeted AAV-DJ-hCBS therapy restores hepatic CBS expression, thereby normalizing systemic homocysteine levels and preventing phenotype manifestation in CBS-deficient mice.

### 2.2. AAV-DJ-hCBS Restores Liver CBS Expression and Limits Inflammation

**Figure 2:**
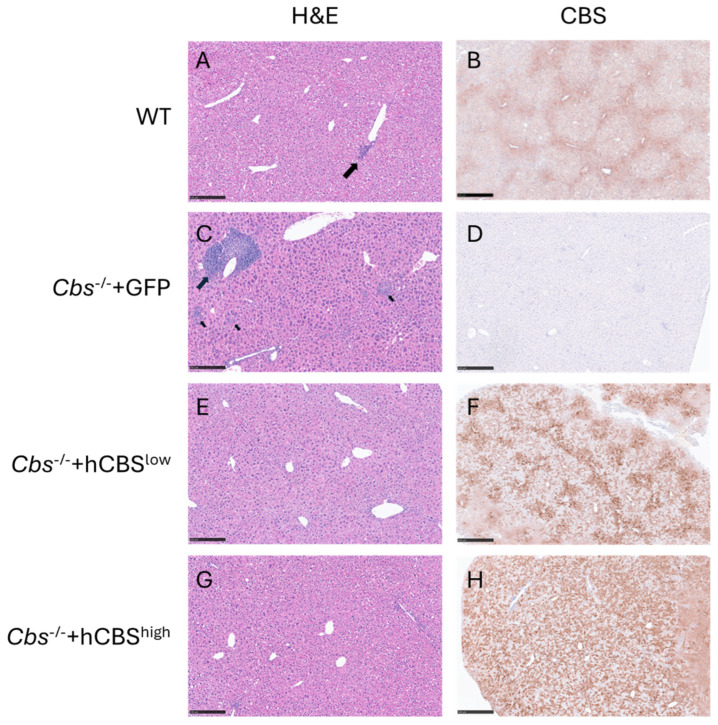
Representative images of liver histology and CBS immunohistochemistry. Paraffin-embedded liver sections were stained with hematoxylin and eosin (H&E; left) and anti-CBS (DAB, brown; right). (**A**,**B**) WT control; (**C**,**D**) *Cbs*^-/-^ + GFP; (**E**,**F**) *Cbs*^-/-^ + hCBS^low^; (**G**,**H**) *Cbs*^-/-^ + hCBS^high^. Large arrows indicate perivascular inflammation; small arrows indicate interstitial inflammation. Bars indicate 250 µm.

Because the liver is the primary site of CBS expression and the target of AAV-DJ delivery, we next examined whether hepatic architecture and transgene expression were preserved following treatment. We therefore investigated liver histology and hCBS immunostaining in all groups. Liver histology revealed occasional mild perivascular inflammation in wild-type mice, with otherwise preserved architecture (Figure 2A). These lesions are commonly observed in 12-month-old C57BL/6 mice and are likely related to the normal aging process rather than a pathological condition [27]. Conversely, *Cbs*^-/-^ + GFP livers displayed pronounced perivascular and focal interstitial inflammation (Figure 2C), suggesting either accelerated aging or underlying pathology. The enhanced inflammation was resolved by AAV-DJ-hCBS treatment, as both *Cbs*^-/-^ + hCBS^low^ and *Cbs*^-/-^ + hCBS^high^ mice demonstrated occasional mild perivascular inflammation similar to wild-type controls (Figure 2E,G). The absence of any histological changes in AAV-DJ-hCBS-treated groups relative to wild-type controls further indicates the absence of any detectable pathological effects elicited by the AAV-DJ-hCBS vector.

We next assessed hCBS transgene localization relative to endogenous hepatic CBS by immunostaining. CBS staining in wild-type livers was distributed throughout the lobules, with increased intensity in the perivenular region (Figure 2B). In *Cbs*^-/-^ + GFP, CBS staining was absent (Figure 2D). The livers of *Cbs*^-/-^ + hCBS^low^ mice showed a CBS expression pattern similar to the wild type, with staining throughout the lobules and accentuated in the perivenular region; however, staining intensity exhibited considerable heterogeneity among individual cells, with increased staining around blood vessels (Figure 2F). *Cbs*^-/-^ + hCBS^high^ mice showed strongly increased CBS staining across the entire lobule (Figure 2H), again with considerable heterogeneity among individual cells. CBS staining revealed no clusters of adjacent CBS-expressing cells, suggesting that genomic integration of the vector DNA and clonal expansion, which has been reported at low levels in the liver for the related AAV serotype 2 [28], is unlikely. Moreover, AAV-DJ-hCBS treatment was performed at 12 weeks of age, a stage when hepatocyte proliferation has ceased [29]. Together, these findings indicate that sustained CBS expression is most likely maintained through episomal vector expression rather than genomic integration.

While AAV-DJ-hCBS treatment generally normalized liver histology in CBS-knockout mice, we noticed two exceptions, both of which were included in the quantitative analyses presented before. Mouse 8 in the *Cbs*^-/-^ + hCBS^low^ group exhibited pronounced steatosis without inflammation, with persistent but patchy hCBS transgene expression (Supplemental Figure S1A,B). Mouse 13 in the *Cbs*^-/-^ + hCBS^high^ group showed marked interstitial inflammation and fibrosis accompanied by complete loss of hCBS expression (Supplemental Figure S1D–F) and redevelopment of HHcy. Interestingly, both mice had developed an antibody response against human CBS (Supplemental Figure S4), which persisted until week 48. Therefore, it is likely that a sustained immune response against hCBS contributed to the abnormal histology observed in these two mice. This is supported by the observation that signs of pathology were absent in an additional five mice that also developed measurable, but transient, antibodies against hCBS, which had returned to baseline by week 48.

Nevertheless, the immunogenic risk observed in these mice likely overestimates the risk in human patients. Anti-hCBS antibody formation in CBS-knockout mice reflects a xenogeneic response to a foreign antigen. In human patients receiving gene therapy encoding the native CBS sequence, central self-tolerance is expected to substantially reduce this risk [30,31,32]. Additionally, while capsid-directed immune responses remain a recognized concern for AAV vectors, engineered capsids such as AAV-DJ have been associated with altered antigenic properties and potentially reduced immunogenicity compared to common natural serotypes [33,34], though dedicated clinical data for AAV-DJ specifically remain limited.

Together, our findings demonstrate that targeted AAV-DJ-hCBS restores liver CBS expression and resolves inflammatory changes without detectable hepatic pathology, consistent with the normalization of the phenotype.

### 2.3. Multi-Omics Reveals One-Carbon and Lipid Pathway Disruption, Reversed by Therapy

To further detail the impact of CBS deficiency on cellular and metabolic pathways and assess reversal by AAV-DJ-hCBS treatment, we investigated the metabolome and proteome of liver tissue in the same wild-type (WT; n = 5), *Cbs*^-/-^ + GFP (n = 5), and *Cbs*^-/-^ + hCBS^low^ (n = 8) mice. *Cbs*^-/-^ + hCBS^low^ was selected for molecular analysis based on complete normalization of the phenotype at lower dosage.

**Figure 3:**
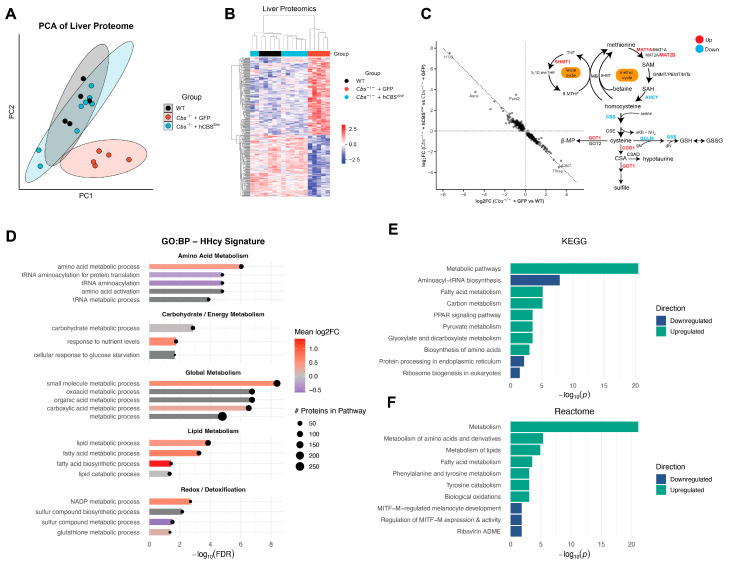
Liver protein signature. (**A**) PCA analysis of all detected proteins. (**B**) Heatmap of significant proteins (ANOVA; FDR < 0.05) with hierarchical clustering. (**C**) Assessing treatment effects by AAV-DJ-hCBS on protein expression in liver. Left: mapping log_2_FC of knockout affected compared to treatment effect in significantly altered proteins. Right: diagram of regulation of detected proteins in the methionine cycle (showing protein expression in *Cbs*^-/-^ + GFP relative to WT; black = not significantly altered; strikethrough = protein was not detected.). (**D**) Top 21 enriched GO: biological processes organized by theme and mapped to their mean log_2_FC in *Cbs*^-/-^ + GFP livers (calculated by taking the mean of all differentially expressed proteins in the specific pathway). (**E**) Top 10 KEGG enriched pathways. Enrichment was separately run on down and upregulated proteins. (**F**) Top 10 Reactome enriched pathways. Enrichment was separately run on down and upregulated proteins.

Across all samples, we identified 3998 proteins, of which 339 were significantly altered in *Cbs*^-/-^ + GFP vs. WT, which were fully restored in *Cbs*^-/-^ + hCBS^low^ to WT levels (Figure 3B,C), signifying the complete normalization of liver protein abundances by AAV-DJ-hCBS treatment. Accordingly, principal component analysis (PCA) of liver proteome demonstrated clustering of WT and *Cbs*^-/-^ + hCBS^low^ which was clearly separated from *Cbs*^-/-^ + GFP mice (Figure 3A).

To explore the signaling routes affected by CBS knockout, we performed pathway enrichment analysis of differentially abundant proteins (DAPs), demonstrating a broad metabolic rewiring in liver of *Cbs*^-/-^ + GFP. A total of 54 biological processes (GO:BP) were significantly altered (Supplemental Table S1), including suppression of tRNA aminoacylation and protein translation pathways and activation of lipid metabolism and redox/detoxification processes (Figure 3D). To distinguish opposing biological programs, we separated DAPs into 148 upregulated and 191 downregulated proteins. Downregulated proteins were enriched for translational and RNA metabolic processes, consistent with a global suppression of protein synthesis. In contrast, upregulated proteins were enriched for lipid, amino acid, and carbohydrate metabolism, consistent with a broad catabolic program (Supplemental Figure S2).

To corroborate these findings across pathway frameworks, we performed enrichment using both KEGG and Reactome. KEGG analysis identified fatty acid metabolism, carbon metabolism, PPAR signaling, and pyruvate metabolism among the most significantly upregulated pathways, whereas aminoacyl-tRNA biosynthesis was suppressed (Figure 3E). Reactome likewise highlighted amino acid, lipid, and tyrosine metabolism, while MITF–M-related transcriptional regulation was downregulated (Figure 3F). Motif enrichment of the upregulated proteins identified HNF4α as the only significantly enriched transcription factor, indicating that the metabolic pathways most affected by CBS deficiency map onto the core hepatocyte program governed by HNF4α.

Collectively, these results demonstrate that AAV-DJ-hCBS therapy normalizes the disease-associated liver proteome, reversing translational suppression and activating catabolic pathways in liver.

**Figure 4:**
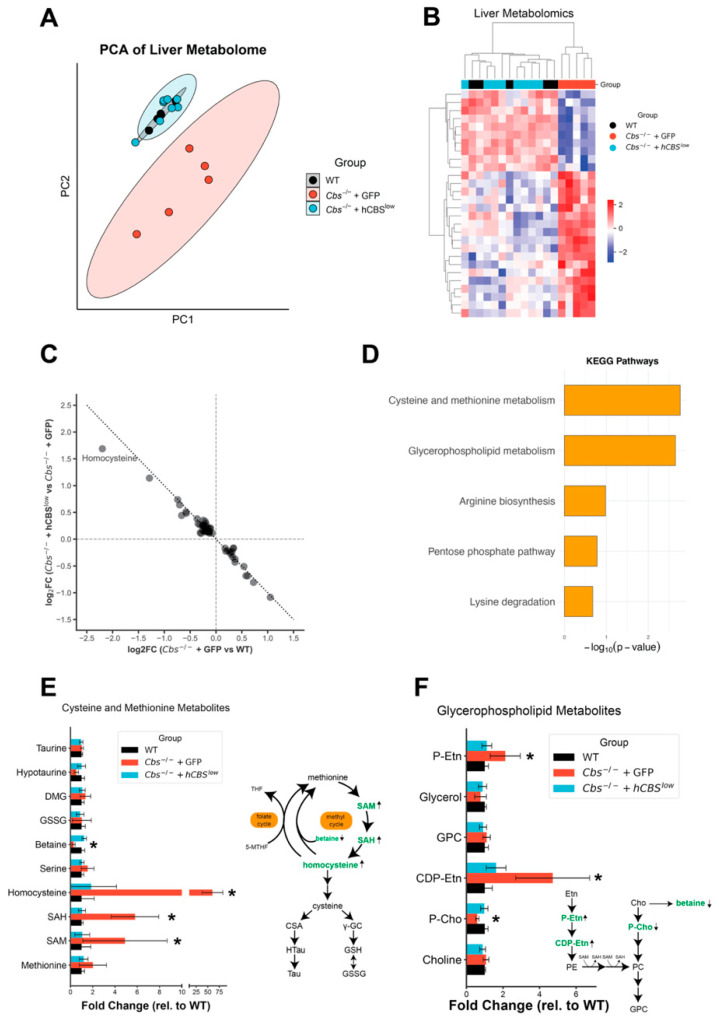
Liver metabolome. (**A**) PCA analysis of all confidently detected metabolites in liver. (**B**) Heatmap of significant metabolites (ANOVA; FDR < 0.05) with hierarchical clustering. (**C**) Assessing treatment effects by AAV-DJ-hCBS on metabolite abundance in liver. Mapping log_2_FC of knockout effect compared to treatment effect in significantly altered metabolites in liver; (**D**) KEGG enrichment with MetaboAnalyst on significantly altered metabolites. (**E**) Detected metabolites in the cysteine and methionine metabolism KEGG pathway. (**F**) Detected metabolites in the glycerophospholipid metabolism KEGG pathway. Bars are represented as mean and standard error. Stars show significance tested by post hoc Tukey analysis.; * *p* < 0.05 vs. WT.

Next, we performed liver metabolomics to further explore rewiring of metabolism in CBS knockout. Across all samples, we detected 993 metabolites, of which 239 were retained for downstream analysis based on high-confidence annotation. AAV-DJ-hCBS therapy normalized the metabolomic profile of *Cbs*^-/-^ + GFP mice, as shown by PCA, which revealed a clear separation between *Cbs*^-/-^ + GFP and WT controls and full overlap of *Cbs*^-/-^ + hCBS^low^ with WT (Figure 4A).

In total, 28 metabolites differed between *Cbs*^-/-^ + GFP and WT, with 18 elevated and 10 reduced, and AAV-DJ-hCBS corrected all to WT levels. This was evidenced by hierarchical clustering, which showed that metabolite levels in *Cbs*^-/-^ + hCBS^low^ were indistinguishable from those in WT mice (Figure 4B), and by normalization of log_2_ fold changes, with homocysteine showing the strongest correction (Figure 4C).

*Cbs*^-/-^ + GFP were characterized by substantial increases in methionine cycle intermediates, SAM, SAH, and homocysteine and decreased betaine. Further, *Cbs*^-/-^ + GFP featured alterations in other metabolites related to SAM-dependent methylation, including a decrease in several quaternary amines (stachydrine, trigonelline, carnitine) and alterations in phosphatidylethanolamine methyltransferase pathway (PEMT-pathway) intermediates (phosphocholine, CDP-ethanolamine, O-phosphorylethanolamine) reflecting impaired methylation of phosphatidylethanolamine into phosphatidylcholine. KEGG pathway analysis of significantly altered liver metabolites highlighted cysteine and methionine metabolism and glycerophospholipid metabolism as enriched (Figure 4D).

Collectively, these data show that within the cysteine and methionine pathways, *Cbs*^-/-^ + GFP mice accumulated SAM, SAH, and homocysteine but maintained stable methionine, which reflects an impaired transsulfuration of homocysteine and a compensatory flux through the betaine-dependent remethylation pathway. Nevertheless, the unchanged γ-glutamylcysteine and glutathione levels indicated preserved redox balance. Further, CBS-knockout animals have impaired PEMT-mediated phosphatidylcholine synthesis, likely due to elevated SAH inhibiting SAM-dependent methylation reactions (Figure 4E,F).

Thus, CBS deficiency disrupts hepatic one-carbon and lipid methylation pathways, which are fully restored by AAV-DJ-hCBS. Together with the liver proteomic data, these metabolomic findings demonstrate that CBS deficiency drives coordinated dysregulation of protein and metabolite networks in HHcy livers, all of which are normalized by AAV-DJ-hCBS therapy.

### 2.4. Plasma Proteome Is Minimally Affected by CBS Deficiency, and Systemic Effects Are Driven by Metabolite Disturbances

**Figure 5:**
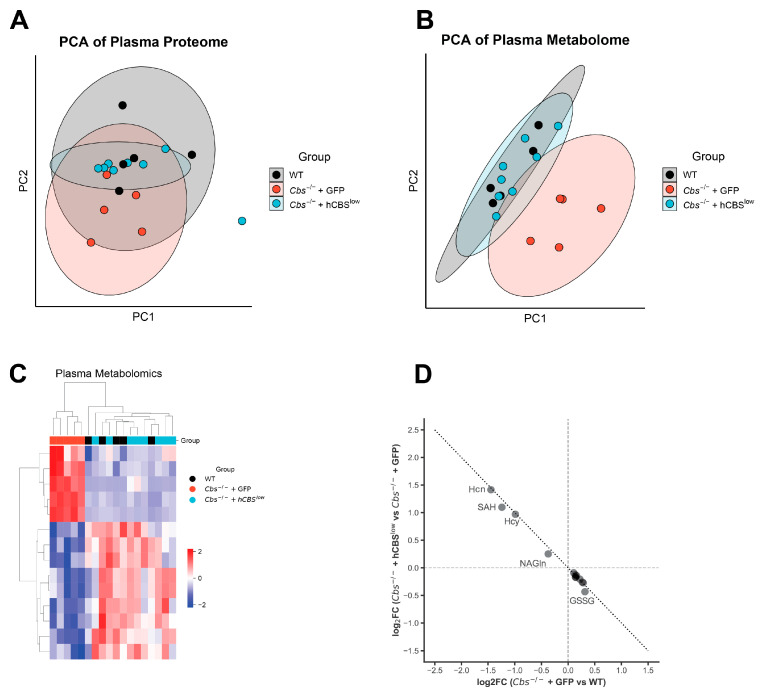
Plasma proteome and metabolome. (**A**) PCA analysis of all detected proteins in plasma. (**B**) PCA analysis of all confidently detected metabolites in plasma. (**C**) Heatmap of significantly altered metabolites (ANOVA; FDR < 0.05) with hierarchical clustering. (**D**) Assessing treatment effects by AAV-DJ-hCBS on metabolite abundance in plasma. Mapping log_2_FC of knockout effect compared to treatment effect in significantly altered metabolites in plasma. Hcn = L-Homocystine; SAH = S-Adenosylhomocysteine; Hcy = Homocysteine; NAGln = N-Acetylglutamine; GSSG = Oxidized glutathione.

Next, we explored whether the impact of *Cbs*^-/-^ was reflected in the blood plasma proteome. LC–MS detected 379 proteins. CBS deficiency did not substantially alter the plasma proteome, and consequently, no proteins differed across groups. As a result, PCA showed strong overlap among WT, *Cbs*^-/-^ + GFP, and *Cbs*^-/-^ + hCBS^low^ mice (Figure 5A). To assess hepatic synthetic function, we curated 84 plasma proteins that are synthesized by the liver and involved in the acute-phase response, lipid and lipoprotein metabolism, coagulation, and complement pathways. Of these, only haptoglobin was increased in *Cbs*^-/-^ + GFP compared with WT and *Cbs*^-/-^ + hCBS^low^, while all other liver-derived plasma proteins not significantly altered. These results suggest minimal impact of CBS deficiency on hepatic plasma protein synthesis and secretion.

In addition, we explored the blood plasma metabolome. In contrast to the proteome, plasma metabolomics revealed systemic alterations in *Cbs*^-/-^ + GFP mice that were fully normalized by AAV-DJ-hCBS. Across all samples, 510 metabolites were detected, of which 217 were retained after high-confidence annotation. PCA showed clear separation of *Cbs*^-/-^ + GFP from WT and *Cbs*^-/-^ + hCBS^low^ (Figure 5B). Thirteen plasma metabolites differed between *Cbs*^-/-^ + GFP and WT, including elevated SAM, homocysteine, and homocystine and reduced isoleucine, choline, taurine, GSSG, glucosamine, and hexose in *Cbs*^-/-^. Hierarchical clustering confirmed complete normalization in *Cbs*^-/-^ + hCBS^low^ (Figure 5C). CBS deficiency perturbs metabolites in a compartment-specific manner, with systemic depletion of redox-related metabolites and branched-chain amino acids in plasma and localized alterations in amino acid modification and mitochondrial function in the liver. Plasma metabolites altered by CBS deficiency included choline, taurine, oxidized glutathione (GSSG), and isoleucine, whereas liver-specific changes affected N-acetylated amino acid derivatives (N-acetylhistidine, N-acetylornithine, N^6^-acetyllysine), carnitine, and 3-hydroxyproline.

Although direct folate and vitamin B6 species were not detected by LC-MS, 4-pyridoxic acid, the primary catabolite of vitamin B6, was detected but not significantly altered (*p* = 0.07), suggesting preserved vitamin B6 catabolism under CBS deficiency.

## 3. Discussion

In this inducible adult-onset *Cbs*^-/-^ mouse model, we observed the anticipated phenotype of severe HHcy (~200 µM), growth retardation, reduced adiposity, and alopecia. Liver-targeted AAV-DJ-hCBS gene therapy was well tolerated and provided sustained correction of HHcy together with rescue of these phenotypic abnormalities. Multi-omics profiling revealed extensive hepatic metabolic remodeling in untreated *Cbs*^-/-^ mice, including suppression of tRNA aminoacylation and protein translation pathways, activating lipid and carbon metabolism, and marked disruptions in methyl-cycle intermediates. Unlike prior AAV-based CBS rescue studies that primarily focused on homocysteine lowering [11], our data demonstrate that selective hepatic CBS restoration is sufficient to re-establish systemic methylation flux and fully reverse coordinated metabolic remodeling in both the liver and plasma. These findings identify the liver as the dominant metabolic control point in CBS deficiency.

The extensive hepatic metabolic remodeling observed in *Cbs*^-/-^ mice suggests that phenotype development is not solely a consequence of homocysteine toxicity or secondary disturbances in glutathione metabolism. Instead, our findings point to dysregulation of the methylation cycle, as evidenced by the accumulation of SAH and SAM. It is well established that SAH inhibits SAM-dependent methyltransferases at multiple levels [35,36,37]. In our dataset, this is reflected by reduced levels of glycerophospholipids, whose synthesis critically depends on SAM-mediated methylation of phosphatidylethanolamine (PE) to phosphatidylcholine (PC) by the enzyme phosphatidylethanolamine N-methyltransferase (PEMT) [38,39,40,41]. Consistently, we previously demonstrated reduced hepatic acyl-carnitines in *Cbs*^-/-^ mice [42], which derive from SAM-dependent methylation of trimethyllysine [38]. At the transcriptional level, reduced methylation is further supported by the enrichment of a HNF4A signature among upregulated proteins, in line with prior evidence that HNF4A expression can be increased through promoter demethylation [43,44,45]. Finally, at the translational level, we observed downregulation of tRNA metabolism and aminoacylation, processes dependent on methyltransferases such as NSUN2, whose loss destabilizes tRNAs and impairs aminoacylation efficiency [34,35,36,37].

The role of methylation-driven alterations in hepatic metabolism of *Cbs*^-/-^ mice is further supported by observations from other methyl-cycle knockout models, which display related biochemical disturbances. SAM synthase-deficient Mat1a^-/-^ mice show reduced hepatic SAM, impaired PEMT activity, and an altered PC/PE balance [46,47,48]. Further, GNMT-/- mice, which accumulate excess SAM due to loss of glycine N-methyltransferase, exhibit similar disturbances, including disrupted methyl flux with global DNA hypomethylation and altered carnitine and acylcarnitine metabolism [49,50,51,52]. Finally, AHCY^-/-^ mice which are deficient for SAH hydrolase (SAHH) are embryonic lethal due to SAH-mediated inhibition of methyltransferases [53], a phenotype comparable to the embryonic lethality of *Cbs*^-/-^ mice [54]. In contrast, the macrocytic anemia we observe in mice appears unique to CBS deficiency, likely reflecting diversion of folate pools into remethylation of homocysteine at the expense of thymidylate biosynthesis, resulting in impaired DNA synthesis and erythropoiesis [55,56,57]. Macrocytic anemia has only been reported in one other mouse study of *Cbs*^-/-^. The absence of this phenotype in our previous study [26] likely reflects differences in genetic background; that model was maintained on a mixed 129/SvEv × C57BL/6 background, in which strain-specific differences in erythrocyte parameters [58] and residual 129-derived genomic regions [59] may have masked this phenotype.

In humans, several case reports have shown macrocytic anemia in classical homocystinuria that could be corrected upon folate supplementation [60,61,62].

Mechanistically, SAH-driven hypomethylation and HNF4α-biased catabolic signaling converge on hepatic lipid handling and whole-body nutrient partitioning. PEMT-mediated phosphatidylcholine (PC) synthesis requires three methyl transfers and is directly inhibited by SAH, limiting PEMT flux, lowering the hepatic PC:PE ratio, and impairing VLDL assembly/secretion mechanisms tightly linked to steatohepatitis in mice and humans [63,64,65,66,67]. In parallel, HNF4A activation promotes fatty acid oxidation and acylcarnitine metabolism, enabling redistribution of fuels to peripheral tissues [68,69]. These shifts are consistent with a protein-sparing, catabolic state, supported by reduced circulating branched-chain amino acids under fasting/protein restriction [70]. Collectively, the hepatic and systemic metabolic changes provide a mechanistic basis for the hallmark phenotypes of CBS-deficient mice, including growth retardation, alopecia, and diminished fat mass [71,72,73]. Notably, selective restoration of CBS expression in the liver is sufficient to normalize these outcomes, even in the context of persistent extrahepatic deficiency, underscoring the liver as the central hub linking methylation flux to systemic metabolic homeostasis [11].

The sole restoration of liver CBS expression was sufficient to normalize systemic biochemical and organismal phenotypes. Despite persistent extrahepatic CBS loss in this model, systemic metabolic homeostasis is maintained, indicating dominance of hepatic methylation control.

More broadly, our findings support reframing hyperhomocysteinemia as a disorder of disrupted methylation flux rather than direct homocysteine toxicity, with SAH as the proximal lesion linking sulfur amino acid metabolism to systemic metabolic dysregulation.

This raises the possibility that restoring systemic methylation balance through liver-targeted CBS therapy could also mitigate complications in other conditions where hyperhomocysteinemia is observed, such as non-alcoholic fatty liver disease [74,75,76,77] and cardiovascular disease [78,79,80]. At the same time, it will be important to assess tissue-specific responses, as SAH accumulation may induce distinct gene programs—such as HNF4A activation in liver—through methylation imbalance. Whether hyperhomocysteinemia in humans likewise reflects primarily a disorder of methylation flux remains an open question. Clinical observations suggest that SAH more strongly correlates with cardiovascular disease than homocysteine [81], and emerging evidence indicates that this relationship may also apply to liver disease [82]. Definitive evidence, however, will require mapping locus-specific methylation changes and linking them to tissue dysfunction in patients. Together, these findings support the view that hyperhomocysteinemia is best understood as a methylation disorder, with SAH as the proximal lesion connecting one-carbon metabolism to systemic disease.

## 4. Materials and Methods

### 4.1. AAV Vector Design and Production

Recombinant AAV vectors encoding human CBS (AAV-DJ-hCBS) or GFP were produced in HEK293F (ThermoFisher, Waltham, MA, USA) cells by triple-plasmid transfection using pHelper, pRep/Cap (AAV-DJ) [22], and transgene plasmids at standard mass ratios. Transfections were performed using FectorVIR (101000004, Polyplus, SA, France), and cells were harvested 72 h post-transfection. AAV particles were released by detergent lysis (1× PBS, 1 mM MgCl_2_, 0.5% Triton X-100; ThermoFisher, Waltham, MA, USA) and purified on an ÄKTA pure 25 system (Cytiva, Marlborough, MA, USA), followed by CsCl ultracentrifugation to deplete empty capsids. Final vector preparations were formulated in buffered solution and stored at −80 °C.

### 4.2. Animal Treatment

All animal experiments and protocols were conducted in accordance with applicable regulations and were approved by the Institutional Animal Care and Use Committee of the University of Groningen (IVD), as well as the Dutch Central Authority for Scientific Procedures on Animals (CCD; AVD1050020186924, approved on 1 October 2019). *Cbs*^fl/fl^ mice were generated as previously described [26] and subsequently backcrossed for at least 10 generations onto a C57BL/6 genetic background. Mice were bred in the animal facility of the University Medical Center Groningen (UMCG) and were allowed to acclimatize to the experimental animal facility for at least two weeks. Both sexes were included, and mice were housed individually under conventional conditions with a 12 h light/dark cycle, controlled temperature (22 ± 2 °C), and ad libitum access to food and water. Additional nesting material (cotton pads) was provided to all groups to mitigate potential heat loss associated with the reduced coat quality of mice with alopecia.

Mice received tamoxifen injections (75 mg/kg body weight, i.p.) for five consecutive days starting at 10 weeks of age to induce CBS deletion. At 11 weeks, plasma homocysteine levels were measured, and all CBS-knockout mice exhibited hyperhomocysteinemia within the target range (100–350 µM). At 12 weeks, anaesthetized mice (2% isoflurane in O_2_) received a single intravenous injection via the retro orbital plexus (maximum volume 100 µL) of AAV-DJ-hCBS either 3 × 10^12^ vg/kg; (hCBS^low^), to restore hepatic CBS expression to physiological levels, or at 3 × 10^13^ vg/kg (hCBS^high^) to assess potential effects of supraphysiological expression. Mice in the GFP group received AAV-DJ-GFP at 3 × 10^13^ vg/kg and served as empty vector controls. Age-matched wild-type littermates served as controls (WT) and did not receive tamoxifen or AAV treatment. All treatments were carried out in a randomized order to minimize potential confounding effects.

*Cbs^-/-^* mice were randomized into treatment groups balanced for plasma homocysteine, body weight, and sex (hCBS^low^, n = 9; hCBS^high^, n = 10; GFP, n = 8). Including WT mice (n = 5), a total of 32 mice were used for the experiments. Group sizes were determined by an a priori calculation assuming a 50% reduction in hyperhomocysteinemia in hCBS versus GFP, yielding n = 7 (two-tailed, effect size = 1.667, α = 0.05, power = 0.8), with 1–2 additional mice included to account for potential mortality. All animals were monitored for 48 weeks with serial measurements of plasma homocysteine, body weight, body composition, and external phenotype. Body weight was recorded weekly. Blood samples for homocysteine measurement were obtained via orbital puncture every 4 weeks from weeks 2 to 20 and every 8 weeks from week 20 to week 40. A final homocysteine measurement was conducted on blood drawn by cardiac puncture at termination (week 48). Body composition and external phenotype characterization were assessed concurrently with homocysteine measurements.

Two mice in the *Cbs*^-/-^ + GFP group died of unknown causes at 1 and 37 weeks after injection. One mouse in the *Cbs*^-/-^ + hCBS^low^ group was euthanized at week 47 due to rapid body weight loss (humane endpoint >15% body weight loss). Body composition (fat, lean, and fluid mass) was assessed by minispec LF Series (Bruker, Billerica, MA, USA).

At study termination (48 weeks), mice were anesthetized with isoflurane and euthanized by exsanguination. Blood was collected in EDTA tubes, and organs were harvested for snap-freezing in liquid nitrogen or fixation in 4% buffered formaldehyde.

### 4.3. Homocysteine

Approximately 50 µL of blood was collected from the retro-orbital sinus of mice under isoflurane anesthesia (2%). Samples were collected in EDTA tubes, and plasma was separated by centrifugation at 2500 *g* for 10 min. For automated homocysteine measurement, 15 µL of plasma was diluted in 185 µL of multi-assay diluent (Abbott, Abbott Park, IL, USA) and analyzed by chemiluminescent microparticle immunoassay on an Alinity analyzer (Abbott, Abbott Park, IL, USA). The assay provided a lower limit of detection of 6–8 µM.

### 4.4. Western Blot Analysis

Liver tissue samples were homogenized in ice-cold RIPA buffer (Igepal CA-630, sodium deoxycholate, and 20% SDS in PBS) supplemented with a protease inhibitor cocktail (Roche Diagnostics, Rotkreuz, Switzerland), sodium orthovanadate (Sigma-Aldrich, St. Louis, MO, USA), and β-mercaptoethanol (Sigma-Aldrich, St. Louis, MO, USA). Total protein concentration was determined using the Bradford assay (Bio-Rad Laboratories, Hercules, CA, USA). Equal amounts of protein (25 µg in 25 µL) were resolved by SDS–PAGE (Bio-Rad Laboratories, Hercules, CA, USA) and transferred to nitrocellulose membranes using the Trans-Blot Turbo system (Bio-Rad Laboratories, Hercules, CA, USA). Membranes were blocked with 5% skim milk (Sigma-Aldrich, St. Louis, MO, USA) for 20 min and incubated overnight at 4 °C with anti-CBS antibody (CBS D8F2P Rabbit Monoclonal Antibody #14782, 1:1000; Cell Signaling Technology, Danvers, MA, USA). After three washes with TBST, membranes were incubated with HRP-conjugated goat anti-rabbit secondary antibody (1:2000; Dako, Glostrup, Denmark) for 2 h at room temperature. Chemiluminescent signals were detected using Western Lightning Ultra (PerkinElmer, Waltham, MA, USA). Blots were reprobed with beta-actin antibody (beta-actin C-7 HRP, 1:1000, Santa Cruz sc-47778, Santa Cruz Biotechnology, Dallas, TX, USA), and CBS expression was expressed as the ratio of CBS over beta-actin.

### 4.5. Droplet Digital PCR (ddPCR)

DNA copy number was determined using the QX200 Droplet Digital PCR system (Bio-Rad Laboratories, Hercules, CA, USA). Reactions (22 μL) contained 11 μL 2× ddPCR Supermix for Probes (no dUTP; Bio-Rad Laboratories, Hercules, CA, USA, Cat. No. 10026868), 0.4 μL of forward primer (100 μM, 5′-GCCAAGTGTGAGTTCTTCAAC-3′), 0.4 μL of reverse primer (100 μM, 5′-CGGATGTCGGCTCGATAAT-3′), 0.2 μL of probe (100 μM, 5′-CCTGCGGATGATTGAGGATGCTGA-3′), 5 μL of nuclease-free H_2_O, and 5 μL of DNA template. Droplets were generated with a QX200 Droplet Generator and amplified under the following conditions: 95 °C for 10 min; 40 cycles of 94 °C for 30 s and 60 °C for 1 min; 98 °C for 10 min; 4 °C hold. Droplets were analyzed with a QX200 Droplet Reader, and copy number was calculated using QuantaSoft Analysis Pro version 1.7.4.0917 (Bio-Rad Laboratories, Hercules, CA, USA).

### 4.6. Metabolomics

Sample analysis was carried out by MS-Omics as follows. The analysis was carried out using a Thermo Scientific Vanquish LC coupled to an Orbitrap Exploris 240 MS (Thermo Fisher Scientific, Waltham, MA, USA). An electrospray ionization interface was used as ionization source. Analysis was performed in positive and negative ionization mode under polarity switching. The UPLC was performed using a slightly modified version of the protocol described by Doneanu et al. (UPLC/MS Monitoring of Water-Soluble Vitamin Bs in Cell Culture Media in Minutes, Water Application note 2011, 720004042en). Peak areas were extracted using Compound Discoverer 3.3 (Thermo Fisher Scientific, Waltham, MA, USA). Compound identification was performed at four levels as follows. Level 1: identification by retention times (compared against in-house authentic standards), accurate mass (with an accepted deviation of 3 ppm), and MS/MS spectra. Level 2a: identification by retention times (compared against in-house authentic standards) and accurate mass (with an accepted deviation of 3 ppm). Level 2b: identification by accurate mass (with an accepted deviation of 3 ppm) and MS/MS spectra. Level 3: identification by accurate mass alone (with an accepted deviation of 3 ppm).

For downstream analyses, only compounds annotated at Levels 1, 2a, or 2b were retained. Data were normalized and subjected to multivariate analysis, differential testing, and pathway enrichment. KEGG pathway enrichment was performed using MetaboAnalyst 6.0 (https://www.metaboanalyst.ca, accessed December 2025 through March 2026).

### 4.7. Proteomics

Liver lysates were mixed with LDS loading buffer (Thermo Fisher Scientific, Waltham, MA, USA) to a final protein concentration of 10 µg, incubated for 10 min at 70 °C, and briefly electrophoresed for approximately 5 min at 100 V on 4–12% Bis-Tris gels (Thermo Fisher Scientific, Waltham, MA, USA). After Coomassie staining (Westburg, Leusden, The Netherlands) and destaining with Milli-Q water, the single protein band was excised, diced, and washed sequentially with 30% and 50% (*v*/*v*) acetonitrile in 100 mM of ammonium bicarbonate for 30 min each at 500 rpm, followed by 100% acetonitrile for 5 min at 37 °C. Proteins were reduced with 10 mM of dithiothreitol for 30 min at 55 °C and alkylated with 55 mM of iodoacetamide for 30 min at room temperature in the dark. After dehydration, proteins were digested overnight with sequencing-grade trypsin (5 ng/µL; Promega, Madison, WI, USA) at 37 °C. Peptides were extracted with 75% acetonitrile containing 5% formic acid for 20 min at room temperature, dried under vacuum, and resuspended in 0.1% formic acid to a final concentration of 0.05 µg/µL.

Discovery-based proteomic analyses were performed on an Orbitrap Exploris 480 mass spectrometer (Thermo Fisher Scientific, Waltham, MA, USA) coupled to an Evosep One LC system equipped with a 15 cm × 150 µm, 1.5 µm column (Evosep, Odense, Denmark) using the 30SPD gradient. Mobile phases consisted of 0.1% formic acid in water (buffer A) and 0.1% formic acid in acetonitrile (buffer B). The instrument was operated in positive-ion HRMS1-direct data-independent acquisition (DIA) mode across a 400–1000 m/z range with 16 m/z isolation windows and FAIMS compensation voltages of −45 V and −60 V.

Raw DIA data were processed with Spectronaut v19.1 (Biognosys, Schlieren, Switzerland) using the directDIA workflow against the Mus musculus UniProt database (17,141 entries). Quantification was performed at the MS1 level with and without local normalization.

Duplicate protein identifiers were collapsed to single entries, and proteins with more than 70% missing values across all samples were excluded from further analysis. At the sample level, those with fewer than 30% of total proteins detected were also excluded. Remaining missing values were imputed per protein, and intensities were log_2_-transformed to yield a normalized dataset.

Data were subjected to multivariate analysis, differential testing, and pathway enrichment. Pathway enrichment was carried out in R using the gprofiler2 package, with all detected proteins as background. To evaluate hepatic synthetic function, a curated plasma protein panel was assembled encompassing acute-phase response, lipid and lipoprotein metabolism, coagulation, and complement pathways, based on liver-derived proteins and Reactome pathway sets (“COMPLEMENT,” “COAGULATION,” and “LIPOPROTEIN|CHYLOMICRON”).

### 4.8. Histology and Immunohistochemistry

Liver samples fixed in 4% buffered formaldehyde were embedded in paraffin, and 4 µm sections were prepared using a microtome (HistoCore AUTOCUT, Leica Microsystems, Wetzlar, Germany). Sections were mounted on glass slides (StarFrost, Ahrensburg, Germany), deparaffinized, and rehydrated through a graded ethanol series (100%, 96%, and 70%; 10 min each).

For hematoxylin and eosin (H&E) staining, sections were stained with Mayer’s hematoxylin (5 min; Sigma-Aldrich, St. Louis, MO, USA), rinsed in tap water, counterstained with eosin (5 min), and mounted with DePeX resin (Serva, Heidelberg, Germany).

For Sirius Red staining, sections were deparaffinized, rehydrated, and stained with 0.1% Sirius Red F3B (Merck KGaA, Darmstadt, Germany, Direct Red 80) in saturated picric acid for 1 h, followed by washing in acidified water (0.5% glacial acetic acid), dehydration through 3 changes in 100% ethanol, and mounting with DePeX.

For CBS immunohistochemistry, sections were deparaffinized and subjected to heat-induced epitope retrieval overnight at 60 °C in citrate buffer (pH 6.0; Sigma-Aldrich, St. Louis, MO, USA). After cooling to room temperature and washing in PBS (Klinipath, Duiven, The Netherlands), endogenous peroxidase activity was quenched with 0.3% hydrogen peroxide (30 min; Sigma-Aldrich, St. Louis, MO, USA). Sections were blocked with 10% goat serum in 1% BSA (Dako, Glostrup, Denmark) for 30 min, followed by overnight incubation at 4 °C with rabbit anti-CBS antibody (1:200, Cell Signaling Technology, Danvers, MA, USA, for liver and skin; 1:5000, Proteintech, Rosemont, IL, USA, for kidney) in PBS containing 1% BSA and 1% goat serum. After three PBS washes, slides were incubated for 1 h at room temperature with HRP-conjugated goat anti-rabbit secondary antibody (1:100, Dako, Glostrup, Denmark), developed with DAB chromogen (Abcam, Cambridge, UK) for 10 min, counterstained with hematoxylin (30 s), dehydrated, and mounted with DePeX.

### 4.9. Data Analysis

Due to the nature of the intervention, blinding during treatment administration was not feasible; however, outcome assessment and data analysis were performed by investigators blinded to group allocation. Reduction in plasma homocysteine levels was defined as the primary outcome and served as the basis for sample size calculation. Secondary outcomes comprised phenotypic changes (including body weight and alopecia), as well as molecular alterations assessed by proteomic and metabolomic analyses.

Data integration and statistical analyses were performed in R (version 4.4.2; RStudio version 2025.09.0 + 387) and Python (version 3.12.11). Data preprocessing, normalization, and visualization were conducted using the tidyverse, ggplot2, and ComplexHeatmap packages in R and with pandas, numpy, and matplotlib in Python. Principal component analysis (PCA) and hierarchical clustering were applied to assess multivariate structure and group separation across proteomic, metabolomic, and biochemical datasets. PCA was performed using scaled and centered log_2_-transformed data (FactoMineR and factoextra in R), and cluster dendrograms were generated from Euclidean distance matrices with complete linkage.

Univariate testing was performed using one-way ANOVA or Student’s *t*-test, depending on the experimental design, implemented in both R (stats and car packages) and Python (scipy.stats). *p* values were corrected for multiple testing using the Benjamini–Hochberg false discovery rate (FDR) method, and features with FDR < 0.05 were considered significantly different.

Graphical representations were finalized in R and Python and prepared for publication using Adobe Illustrator version 30.2 (Adobe Inc., San Jose, CA, USA).

All data are presented as the mean ± standard error of the mean, unless otherwise specified.

### 4.10. Generative AI Statement

Large language model-based generative AI tools (ChatGPT, OpenAI, GPT-5.2; accessed 2026) were used for language editing and for debugging data analysis code. No AI tools were used for data generation, analysis decisions, or interpretation. All scientific content and conclusions were verified by the authors.

## Figures and Tables

**Table 1:** Organ weights and blood parameters.

	Wild Type	*Cbs*^-/-^ + GFP	*Cbs*^-/-^ + hCBS^low^	*Cbs*^-/-^ + hCBS^high^
Heart (mg)	175.2 ± 13.5	170.0 ± 47.1	181.3 ± 34.2	175.6 ± 29.7
Left kidney (mg)	199.6 ± 21.5	213.7 ± 34.5	205.2 ± 17.9	211.1 ± 25.3
Right kidney (mg)	221.6 ± 18.1	224.3 ± 23.4	214.7 ± 30.9	230.3 ± 27.7
Spleen (mg)	97.6 ± 28.6	94.3 ± 29.4	88.3 ± 39.4	84.1 ± 17.7
Liver (mg)	1682 ± 166	1591 ± 171	1579 ± 321	1762 ± 408
Brain (mg)	473.0 ± 18.1	464.7 ± 17.8	477.1 ± 24.7	494.8 ± 36.5
Glucose (mM)	13.4 ± 1.2	9.7 ± 1.4 *	12.8 ± 3.3	13.2 ± 0.9
WBC (×10^9^/L)	6.3 ± 1.9	4.2 ± 2.3	6.0 ± 4.6	4.8 ± 1.7
RBC (×10^12^/L)	12.4 ± 1.4	8.4 ± 3.3 *	11.4 ± 1.4	12.1 ± 1.7
HGB (mmol/L)	10.5 ± 1.1	8.9 ± 1.5	9.8 ± 1.1	10.4 ± 1.2
HCT (L/L)	0.63 ± 0.07	0.51 ± 0.10 *	0.58 ± 0.07	0.61 ± 0.08
MCV (fL)	50.6 ± 1.0	51.9 ± 4.0	50.4 ± 0.6	50.5 ± 1.0
MCH (amol)	852.2 ± 28.3	914.4 ± 59.4 *	863.1 ± 21.6	864.5 ± 32.9
MCHC (mmol/L)	16.8 ± 0.3	17.6 ± 0.6	17.1 ± 0.3	17.0 ± 0.6
PLT (×10^9^/L)	1273 ± 186	1240 ± 655	1001 ± 479	1163 ± 372

WBC, white blood cell; RBC, red blood cell; HGB, hemoglobin; HCT, hematocrit; MCV, mean corpuscular volume; MCH, mean corpuscular hemoglobin; MCHC, mean corpuscular hemoglobin concentration; PLT, platelet; represented as mean ± SEM; * *p* < 0.05 vs. WT.

## Data Availability

The raw data supporting the conclusions of this article will be made available by the authors on request.

## Supplementary Materials

The following supporting information can be downloaded at: https://www.mdpi.com/article/10.3390/ijms27073338/s1.

## Abbreviations

The following abbreviations are used in this manuscript:

AAV	Adeno-associated virus
CBS	Cystathionine β-synthase
DAP	Differentially abundant protein
ddPCR	Droplet digital polymerase chain reaction
GFP	Green fluorescent protein
GO:BP	Gene Ontology: Biological Process
GSSG	Oxidized glutathione
H&E	Hematoxylin and eosin
hCBS	Human cystathionine β-synthase
HCT	Hematocrit
HCU	Classic homocystinuria
Hcy	Homocysteine
HGB	Hemoglobin
HHcy	Hyperhomocysteinemia
HNF4α	Hepatocyte nuclear factor 4 alpha
KEGG	Kyoto Encyclopedia of Genes and Genomes
LC–MS	Liquid chromatography–mass spectrometry
MCH	Mean corpuscular hemoglobin
PC	Phosphatidylcholine
PCA	Principal component analysis
PE	Phosphatidylethanolamine
PEMT	Phosphatidylethanolamine N-methyltransferase
PLP	Pyridoxal 5′-phosphate
RBC	Red blood cell
SAH	S-adenosylhomocysteine
SAM	S-adenosylmethionine
WT	Wild type
