# Supplementary material for: Liver-Targeted AAV-DJ-hCBS Therapy Achieves Long-Term Correction of Metabolic Imbalance in CBS-Deficient Mice
Source: Int J Mol Sci. 2026 Apr 7;27(7):3338. doi: 10.3390/ijms27073338 (PMC13073810; doi:10.3390/ijms27073338)
Supplement: Supplementary file 1 [file ijms-27-03338-s001.zip › ijms-4222524 Supplementary Figures-done.pdf]

International Journal of
Molecular Sciences

MDPI

Supplemental Figures

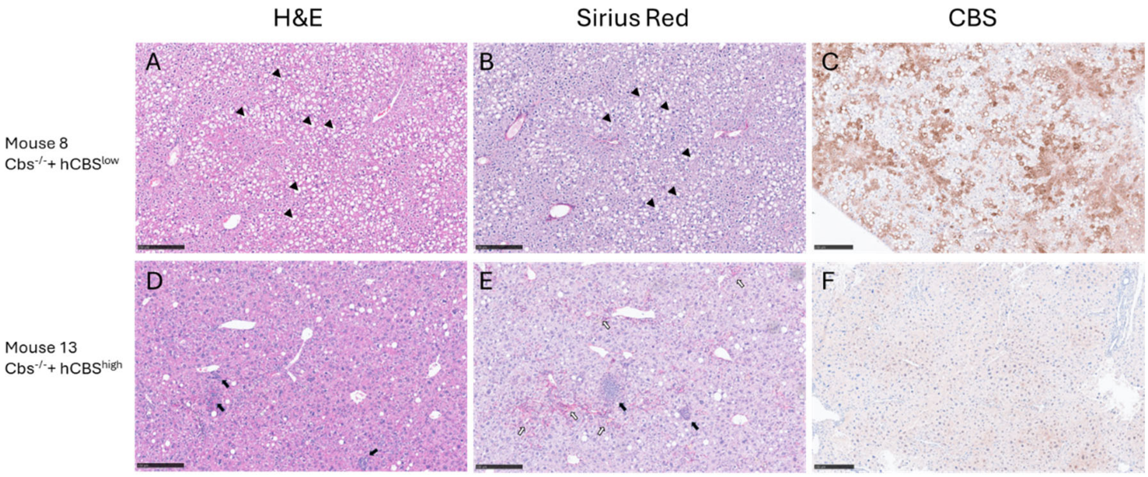

Figure S1. Mice with anti-hCBS antibodies. Liver histology of mice showing anti-human CBS antibodies. (**A,D**) HE staining, (**B,E**) Sirius red and (**C,F**) CBS immune staining of mouse 8 and 13 respectively. Small arrows indicate interstitial inflammation, white arrows indicate interstitial fibrosis, arrowheads point to lipid vesicles. Bar indicates 250 µm.

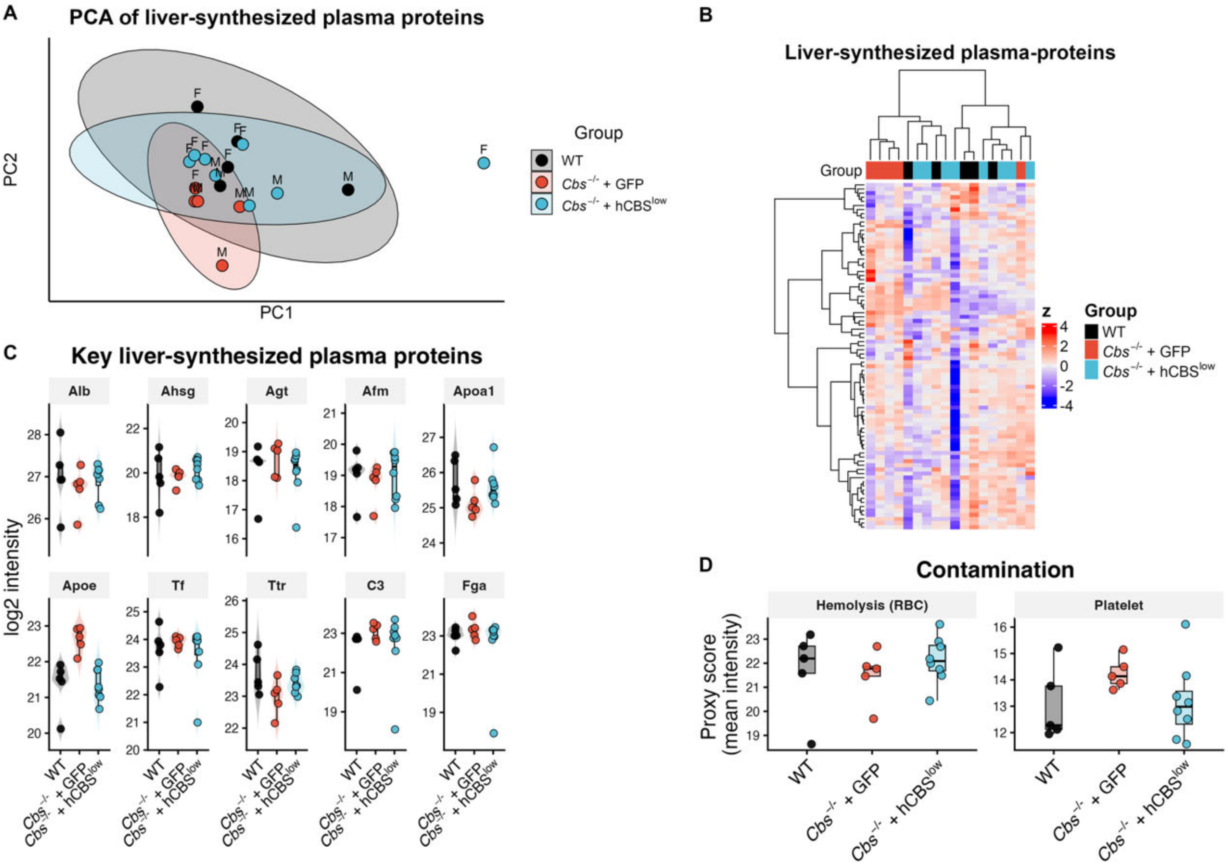

Figure S2. Analysis liver-synthesized plasma proteins. (**A**) PCA of a curated set of liver synthesized plasma proteins (acute-phase, transporters, lipoproteins, complement and coagulation). (**B**) Hierarchically clustered heatmap of liver-synthesized plasma proteins showing z-scored abundance of proteins. (**C**) Violin plot showing distribution of selected liver-derived plasma proteins with boxplots and individual points. (**D**) Assessment of possible hemolysis or platelet contamination in

plasma proteins. Boxplots show proxy scores calculated by mean abundance of canonical marker proteins.

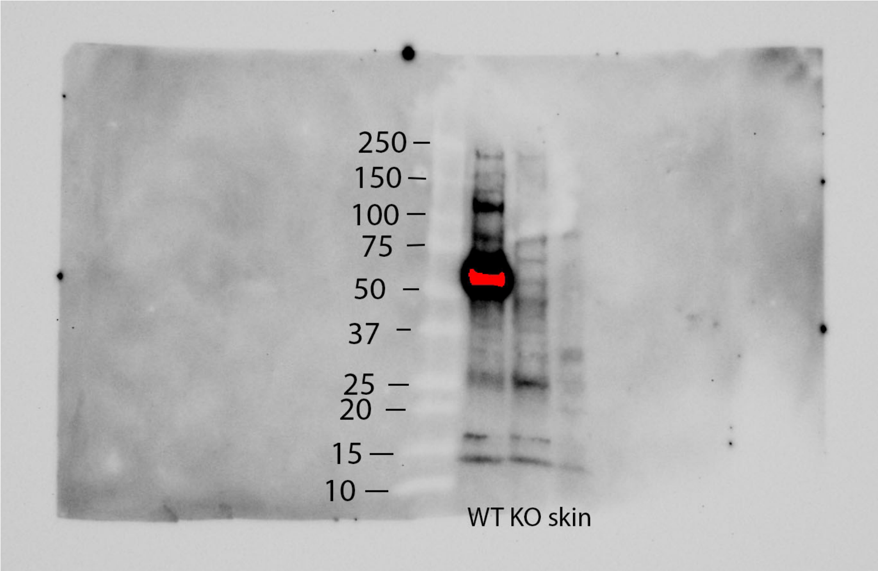

(A)

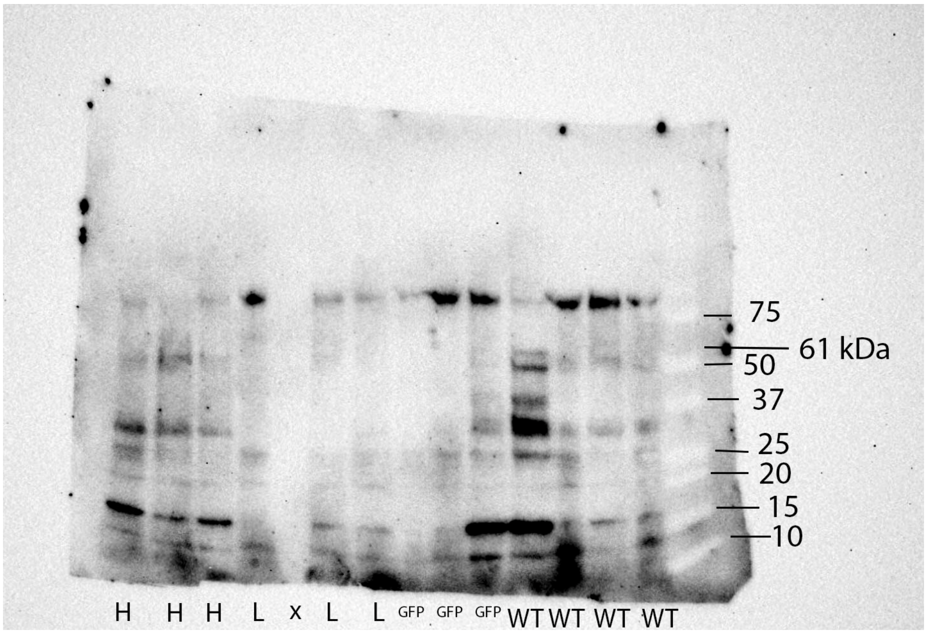

(B)

Figure S3. Western Blot CBS detection in skin of mice. **(A)** CBS-antibody detection at 61 kDA in wild-type (WT) and $Cbs^{-/-}$ (knockout) liver and in wild-type skin (skin). (**B)** CBS-antibody detection in skin of wild-type (WT), $Cbs^{-/-}$+GFP (GFP), $Cbs^{-/-}$+hCBS^{low} (L), and $Cbs^{-/-}$+hCBS^{high} (H).

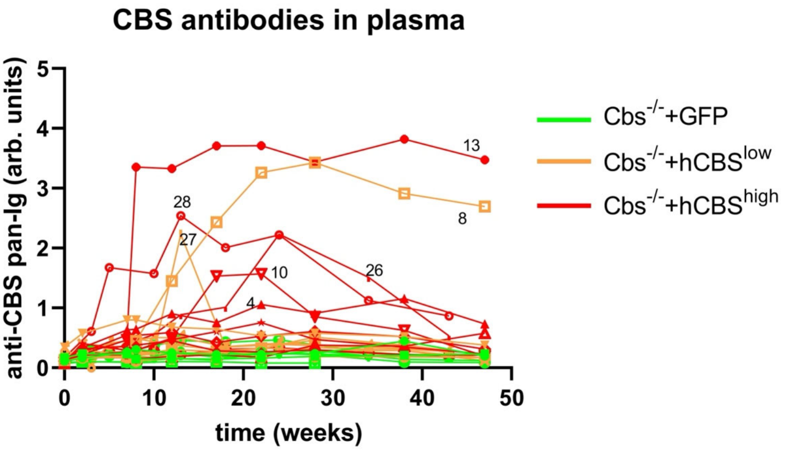

Figure S4. Anti-hCBS antibodies in plasma over time.
